# Generation of High Dose Inhalable Effervescent Dispersions against Pseudomonas aeruginosa Biofilms

**DOI:** 10.1007/s11095-020-02878-w

**Published:** 2020-07-19

**Authors:** Aram Mohammed, Jakub Zurek, Somto Madueke, Hareir Al-Kassimy, Muhammad Yaqoob, Chahinez Houacine, Amina Ferraz, Rachith Kalgudi, Mohammed Gulrez Zariwala, Nicholas Hawkins, Hisham Al-Obaidi

**Affiliations:** 1 The School of Pharmacy, University of Reading, Reading, RG6 6AD UK; 2 Interaction Chempharm Ltd, Reading, RG2 0QX UK; 3 School of Pharmacy and Biomedical Sciences, University of Central Lancashire, Preston, PR1 2HE UK; 4 School of Life Sciences, University of Westminster, 115 New Cavendish Street, London, W1W 6UW UK; 5 Department of Engineering Science, University of Oxford, Parks Road, 0X1 3PJ, Oxford, UK

**Keywords:** ciprofloxacin, Co-amorphous, effervescent, dry powder inhaler, *Pseudomonas aeruginosa*

## Abstract

**Abstract:**

**Purpose:**

Novel particle engineering approach was used in this study to generate high dose inhalable effervescent particles with synergistic effects against *Pseudomonas aeruginosa* biofilms.

**Methods:**

Spray dried co-amorphous salt of ciprofloxacin (CFX) and tartaric acid (TA) was prepared and coated with external layer of sodium bicarbonate and silica coated silver nanobeads. Design of experiments (DOE) was used to optimize physicochemical properties of particles for enhanced lung deposition.

**Results:**

Generated particles were co-amorphous CFX/TA showing that CFX lost its zwitterionic form and exhibiting distinct properties to CFX/HCl as assessed by FTIR and thermal analysis. Particles exhibited mass mean aerodynamic diameter (MMAD) of 3.3 μm, emitted dose of 78% and fine particle dose of 85%. Particles were further evaluated via antimicrobial assessment of minimum inhibitory concentrations (MIC) and minimum biofilm eradication concentration (MBEC). MIC and MBEC results showed that the hybrid particles were around 3–5 times more effective when compared to CFX signifying that synergistic effect was achieved. Diffusing wave spectroscopy results showed that the silver containing particles had a disruptive effect on rheological properties as opposed to silver free particles.

**Conclusions:**

Overall, these results showed the potential to use particle engineering to generate particles that are highly disruptive of bacterial biofilms.

## Introduction

Biofilms are highly dense collection of microbial cells embedded within a self-produced matrix of exopolysaccharide (EPS) (1). The composition of this bacterial matrix can significantly vary but generally it is composed of a polysaccharide rich structure with proteins and DNA (2,3). While this multilayer structure is considered resistant to penetration of antibiotics, it is equally efficient for water and nutrients transports for the survival of the bacteria (4). Multiple studies have examined the resistance pathways of bacterial biofilms, but to date there is no effective technology on how to overcome this mechanism of drug impedance (5). *Pseudomonas aeruginosa* is a Gram-negative bacteria responsible for causing a wide range of nosocomial infections, most notably in immunocompromised patients. Its ability to form robust biofilms as part of the infection renders them chronic and difficult to treat hence, there is an urgent need for synergistic antibiotic therapies (6).

Pulmonary drug delivery using dry powder inhalers (DPIs) has been widely used to manage respiratory conditions (7). However, the main challenge with using DPIs is the difficulty to achieve high levels of the drug inside the lungs (8,9). The low drug deposition is due to loss of the drug in the throat as well as in the device itself. Different strategies have been used to improve the flow properties of the drug particles such as the use of lactose carrier particles (10–12). This approach is based on blending the drug particles with the larger carrier particles which separate inside the device in response to the high inspiratory flow rate generated by the patient (12,13). However, using carrier particles means the mass of the drug is significantly diluted hence this approach cannot be used to deliver large drug doses. In order to avoid the use of carrier particles, the drug molecules are combined with co-former molecules to form co-amorphous dispersions. This approach was used in this study to form of co-amorphous salts dispersions where the drug and the co-former interact as twin molecules forming a glassy solid solution. Advantage of this approach is the possibility to tailor the properties of the final particles to target a localised region within the air conducting zone inside the lungs.

**Fig. 1:**
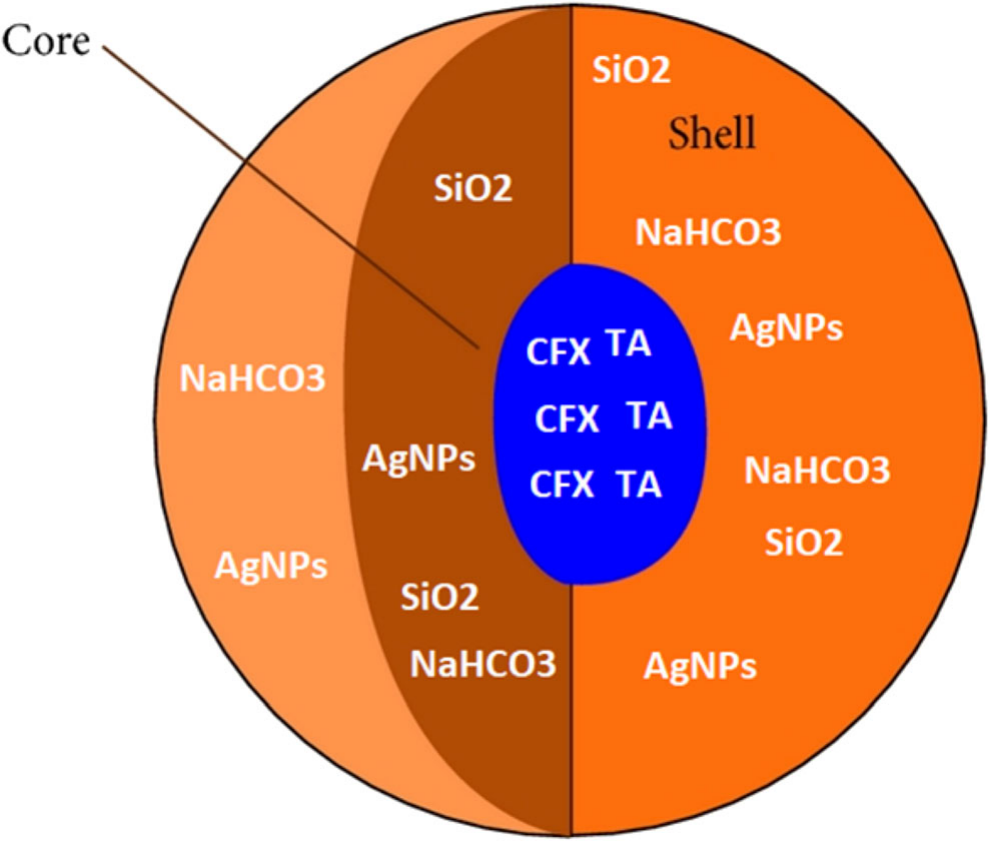
Schematic showing the structure of prepared microparticles. The shell incorporates the silica coated silver nanoparticles and NaHCO_3_ while the core comprised of the CFX/TA co-amorphous salt.

Dental plaques are examples of resistant bacterial biofilms that adhere to the surface via enhanced viscoelastic properties (14). The use of effervescent tablets was found more effective at removing dental plaque than brushing only (15). Hence, the use of effervescent particles can be effective method to disrupt bacterial biofilms. Synergism can also be achieved using silver nanoparticles due to their antibacterial and mucolytic properties (16). Ciprofloxacin (CFX) is a poorly water-soluble drug with broad antibacterial activity and is the drug of choice for treatment of *P. aeruginosa* infections. Currently, there are no inhaled formulations of CFX due to the high doses needed to achieve therapeutic levels. The aim of this study is to use an aliphatic acid (tartaric acid) as a co-former to formulate co-amorphous salt employing spray drying to produce particles with improved flow properties and synergistic effects against *Pseudomonas aeruginosa*. These will be combined with silver coated silica beads and external layer of sodium bicarbonate to produce effervescent effect (Fig. 1). Tartaric acid has been shown to produce central nervous system-mediated bronchodilatation (17) while organic acids in general have been shown to exhibit bacteriostatic activity (18). Using design of experiments (DoE), spray drying conditions were optimised to generate co-amorphous particles. The formed particles were then analysed to assess aerodynamic parameters, viscoelastic properties and antimicrobial activity.

## Material and Methods

### Materials

CFX (Ciprofloxacin) powder ≥98.0% (HPLC), (CAS: 85721–33-1) was purchased from Sigma-Aldrich (Dorset UK). TA (L-(+)-Tartaric acid) crystalline powder, ≥99.7%, CAS: 87–69-4, was purchased from Sigma-Aldrich (Dorset UK). Ethanol (99.8%) of analytical grade was obtained from Sigma-Aldrich (Dorset UK). Tetraethyl orthosilicate (TEOS; ≥99.0%, GC, Sigma-Aldrich), silver (Ag; nanopowder, <100 nm particle size, 99.5% trace metals basis, Sigma-Aldrich), dimethylamine (DMA) and sodium bicarbonate from Sigma-Aldrich. Ultrapure water or high purity lab water (HPLC gradient grade) was obtained from Fisher Scientific UK. All other chemicals used were of analytical grade unless otherwise stated. *P. aeruginosa* PAO1 (wild type) and *P. aeruginosa* NCT10662 were obtained from The University of Westminster (London, UK) culture collection. Bacterial strains were all cultured at 37°C. Luria-Bertani broth and agar (LBB and LBA, Sigma-Aldrich, Dorset, UK) were used for determination of colony count for biofilm cell viability assay.

### Preparation of Co-Amorphous Salt Dispersions Using Optimised Spray Drying Conditions and Incorporation of Silica Coated Silver with NaHCO_3_

Spray-drying experiments were carried out using a spray dryer B-290 from Büchi (Laboretechnik AG Switzerland) using nitrogen gas as the drying gas. Mixtures of CFX (API) with TA (co-former) were prepared using different molar ratios; 1:1, 1:2, 1:3 and 2:1 with the total weight of 1 g and. The solvents that were used to prepare the solutions were 90% water: 10% ethanol, 80% water: 20% ethanol, 70% water: 30% ethanol, 60% water: 40% ethanol and 100% water. The solutions were then spray dried using an airflow of 400 L/h, feed rate 10 mL/min and inlet temperatures 100°C, 120°C, 140°C. The aspiration level was set at 100% and the pump rate at 8 mL/min and 10 mL/min. The resulting outlet temperature ranged from 60 to 75°C. The spray-dried product (powder) was collected from the cyclone collector of the spray dryer and stored in a vacuum desiccator on silica gel for further analysis. Samples were then collected and stored under 0% relative humidity and ambient conditions. Silica coated silver nanobeads were prepared following similar method described before (16). Briefly, 75 mL of ethanol (absolute), 20 mL distilled water and silver nanoparticles (1 mg) were mixed followed by addition of 1 mL dimethylamine combined with ultra-sonication. 1 mL TEOS were added gradually whilst on vigorous stirring using mechanical stirrer. The silica coating started almost immediately which was observed by increased turbidity of the suspension signifying formation of the silica around the suspended silver nanoparticles. The formed suspension of the formed silica coated silver nanoparticles was added to NaHCO_3_ solution (prepared using equimolar concentration to TA) and spray dried simultaneously using three-fluid nozzle with the CFX/TA and using similar spray drying conditions described above. The solutions were kept stirring during spray drying process to prevent any precipitation.

### Powder X-Ray Diffraction (XRPD)

The XRPD pattern scans of pure CFX and TA and all spray dried samples were collected using a Bruker D8 advance X-ray diffractometer (Bruker AXS GmbH, Germany). The x-ray beam is based on a Cu-source, theta diffractometer equipped with a Lynx eye position sensitive detector. Brunker D8 Advance was operated at 40 kV generator voltages and 40 mA generator current. The samples were analyzed using DFFRAC plus XRD commander software (Bruker AXS GmbH, Germany) with a 2θ range of 5–45°, a step size of 0.021064112° and time per step of 1.33 s.

### Thermal Analysis Using Differential Scanning Calorimetry (DSC)

Thermal analysis of the co-amorphous dispersion was performed using differential scanning calorimetry (DSC Q2000, TA instruments, UK). Samples were placed in a crimped aluminium pan before being hermetically sealed. A typical thermogram was obtained by initially allowing the sample to equilibrate at 20°C for 2 min. Samples were then heated up to 300°C at a heating rate of 10°C/min. All samples were purged with nitrogen gas flowing at 50 mL/min. The thermograms that were produced were then analysed using Universal Analysis 2000 (TA instruments).

### Fourier Transforms Infrared Spectroscopy (FTIR)

FTIR was carried out to determine molecular interactions between CFX and TA in each spray dried powder samples. Perkin Elmer Spectrum One FTIR spectrometer was used to record infrared spectra of all samples. For each sample, 16 scans were carried out using a resolution of 4 cm^−1^ and the scanning mode was set between the frequency range of 4000 cm^−1^ to 40 cm^−1^.

### Kinematic Viscosity Measurements

The viscosities of solvent mixtures and solutions were measured using iVisc capillary U-tube viscometer (viscometer constant = 0.001) (Lauda, Königshofen, Germany). Sample solutions were filled into one side of the viscometer and the time taken to reach the labelled point was measured over time. Average values of a minimum three measurements were recorded.

### Microrheology Using Diffusing Wave Spectroscopy (DWS)

Simulation of particles interactions with model biofilm liquid was studied using glycerol to mimic the biofilm physical properties. Samples were measured in the Tau Lag and Echo modes using LS Instruments DWS Rheolab (LS Instruments, Switzerland). Samples were loaded into quartz cuvette and the Tau lag mode captures data over the higher measurement frequencies. Tau lag mode captures information about the sample over short relaxation timescales whereas the Echo mode captures the sample response at longer relaxation periods (Lower measurement frequencies). This implies that measurement of the longer relaxation processes can be collected rapidly (typically 30–60 Seconds). Ultimately, measurement of the loss modulus, storage modulus (G’ and G””) and complex viscosity were obtained.

### Analysis of Powder Deposition Using Anderson Cascade Impactor (ACI)

Anderson Cascade Impactor (ACI; Copley Scientific, Nottingham, UK) was used to analyse the deposition pattern of DPI formulations. All capsules had the same amount of CFX dose of 30 mg and were filled in HPMC size 3 capsules which were loaded in a RS01 DPI device prior to testing. Flow meter was attached to the induction port to verify a flow rate of 60 L/min. Once flow rate of 60 L/min was achieved the RS01 DPI device was connected to the induction port which was then followed by releasing the dose from the device. The cut-off sizes for the ACI plates using 60 L/min as from stage 1 to stage 8 were as follows: 8.6, 6.5, 4.4, 3.3, 2, 1.1, 0.54 and 0.25 μm. After each run, each ACI stage plate was rinsed with 30 mL 3% acetic Acid. Concentration of CFX was then analysed using UV spectrophotometer (Jenway, Staffordshire, UK) at 278 nm absorbance. All experiments were conducted in triplicate.

### Scanning Electron Microscopy (SEM)

Samples were fixed on to the surface of a conductive double-sided carbon adhesive, attached to an aluminium stub. The prepared samples were sputter coated with gold, for 3 min at 30 mA, using Emitech K550 (Ashford, UK). The micrographs were collected using a Philips FEI microscope (Eindhoven, Netherlands).

### Determination of Minimum Inhibitory Concentrations and Minimum Biofilm Eradication Concentration

Minimum inhibitory concentrations (MIC) and minimum biofilm eradication concentration (MBEC) of the dispersions were determined against *P. aeruginosa* PAO1 and *P. aeruginosa* NCTC 10662 strains using a broth dilution method. The results obtained were interpreted according to guidelines set by European Committee on Antimicrobial Susceptibility Testing guidelines. MBEC of the formulations was determined by the method described by Rudilla et al. with a few modifications (19). Briefly, pegs with a modified polystyrene lid (Nunc Immuno TSP system) were immersed into a separate 96-well microtiter plate containing 200 μL of LBB inoculated with *P. aeruginosa* PAO1 and *P. aeruginosa* NCTC 10662 per well, respectively. The plates were then incubated at 37°C for a period of 24 h under static conditions. The pegs were then rinsed with 0.5 M PBS solution to remove unattached cells and immersed into a 96-well plate containing CFX samples and then incubated again at 37°C for a period of 24 h under static conditions. Bacterial cells from the biofilm were extracted by sonication for 10 min after an initial wash with 0.5 M PBS. The optical density of the recovered bacterial cells was measured at 600 nm and the data was used to determine the MBEC. The lowest concentration of the formulation that prevented the growth of bacterial cells extracted from the treated biofilm was used to determine the MBEC. All experiments were performed in triplicates.

### Biofilm Bacteria Enumeration after MIC Treatment

Biofilms of *P. aeruginosa* PAO1 and *P. aeruginosa* NCTC 10662 were grown as mentioned under biofilm quantification by microtiter plate assay. The microtiter plates were incubated for 4 h and 12 h at 37°C. After aspiration of planktonic cells and spent medium, 200 μL of 0.5 M PBS was added to each well and sonicated for 10 min. On pre-prepared LBA plates, 100 μL of recovered cells were plated and incubated overnight at 37°C and colonies were counted the following day.

### Microtiter Plate Assay for Biofilm Quantification

Biofilms of *P. aeruginosa* PAO1 and *P. aeruginosa* NCTC 10662 were formed on 96-well flat bottom polystyrene micro-titre plates. Bacterial cell suspension was adjusted to 0.5 McFarland standard and 10 μL of the cell suspension was inoculated into 190 μL of LBB medium with and without the presence of formulations in their respective wells. The 96-well micro-titre plates were then incubated at 37°C for a period of 18 h. After aspiration of planktonic cells and spent medium, the biofilms were stained with 200 μL of crystal violet solution (0.1%) at room temperature for a period of 5 min. The plates were then gently washed to remove excess crystal violet and airdried. Finally, the biofilm bound crystal violet was dissolved in 33% acetic acid and the solution was measured at O.D 570 nm using a microplate reader (BMG SPECTROstar Nano).

### Pyocyanin and Rhamnolipid Quantification

Pyocyanin quantification was performed based on the method by Essar et al. with some modifications (20). Quantification was based on absorbance of pyocyanin at 520 nm under acidic conditions after phase separation using chloroform. Biofilms were resuspended in 1x PBS, then 500 μL of the suspension was extracted with 3 mL of chloroform and then re-extracted into 2 mL of 0.2 M HCl to provide a pink to red solution. A volume of 200 μL of the solution from the samples was then transferred to a microtiter plate and the absorbance was measured at 520 nm. Quantification of rhamnolipid was performed by applying the orcinol reaction as described by Laabei et al. with modifications (21). Briefly, the supernatant was extracted 3 times with 1 mL diethyl ether prior to completing evaporation under vacuum. Upon completion, 0.5 mL of distilled water was added to each of the sample tubes. For the assay, 100 μL of samples were taken after resuspension in dH_2_O. A volume of 900 μL of 0.19% orcinol (diluted in 53% H_2_SO_4_) was added to the samples and then incubated at 80°C for 30 min. After incubation, the samples were left to cool at room temperature and the absorbance was measured at 421 nm. The concentration was determined based on the standard curve prepared using rhamnose.

### Statistical Analysis

Statistical analysis was carried out using JASP software (v 11.1, Netherlands). Data were analysed using one- and two-sample Student *t* tests, or one-way analysis of variance (ANOVA) and plotted using linear regression and Q-Q analysis. A *p* value of ≤0.05 was considered significant.

## Results

### Preparation of Co-Amorphous Ciprofloxacin Tartarate Using Spray Drying

A mixture of ethanol and water was used to form the silica coated silver nanoparticle. Initial assessment was undertaken to optimise the spray drying conditions of the co-amorphous salt (CFX-TA) to avoid precipitation/crystallization of CFX during spray drying. We identified three parameters that can affect the properties of the particles which were: CFX to TA molar ratio, ratio of ethanol to water in solution prior to spray drying and inlet temperature. CFX is not soluble in ethanol hence ratio of ethanol in the solvent system was optimised to prevent crystallization upon spray drying.

**Fig. 2:**
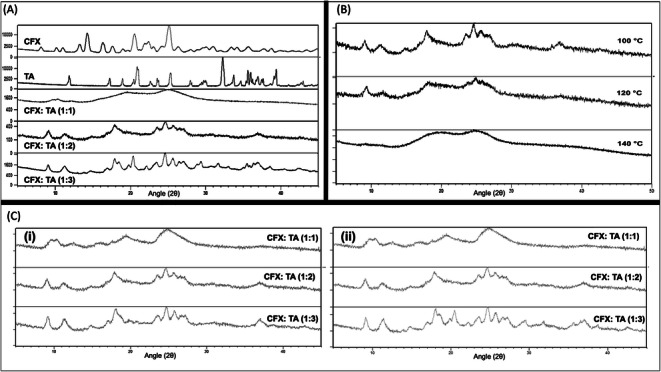
XRPD scans of (A) CFX, TA and corresponding spray dried dispersions of CFX/TA of different molar ratios, (B) spray dried CFX/TA (1:2) dispersions prepared using inlet temperatures of 100, 120, 140°C and (C) spray-dried CFX-TA dispersions prepared using (i) 10% *v*/v ethanol in water solution and (ii) 20% v/v ethanol in water solution.

As shown in Fig. 2, increasing the molar ratio of TA resulted in higher crystallinity. Crystalline peaks could be detected with some amorphous form formation. XRPD showed distinct peaks appearing at 9, 18, 19, 21, 25 and 36° which suggests that the formed crystalline structures are the same regardless of CFX:TA ratio. During spray drying, the temperature of the droplets is increased to near/above the boiling temperature of the solvent. Hence, a temperature range for the spray drying inlet temperature was selected based on the solvents system, which was 100, 120 and 140°C. As can be seen in Fig. 2, using lower inlet temperature led to higher crystalline content with peaks appearing at the same positions mentioned above. When the temperature was increased to 140°C the peaks were less distinct indicating formation of amorphous form. Overall trend revealed that dispersions prepared using 100°C showed highest crystallinity content compared to dispersions prepared using higher inlet temperatures. It is worth mentioning that a higher inlet temperature does not necessarily imply optimised amorphous content as, depending on molar ratio of CFX to TA, this can lead to rapid evaporation of ethanol and separation of excess CFX/TA as separate crystals. As expected, because of low CFX solubility in ethanol, addition of ethanol in the solvent mixture increased CFX crystallinity. However, the impact on crystallinity content was less when compared with the impact of changing the molar ratio of CFX to TA.

**Fig. 3:**
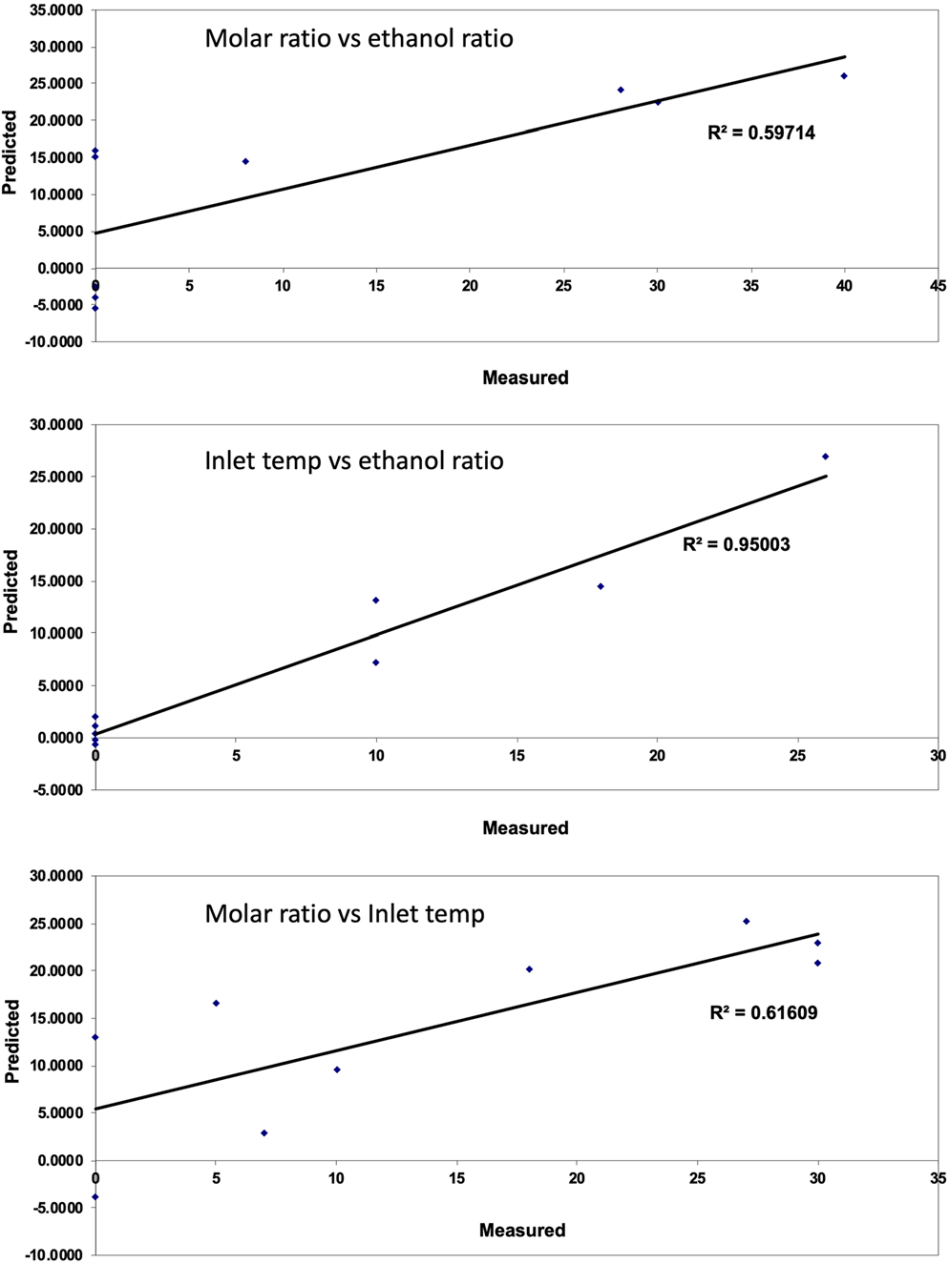
Predicted versus measured crystallinity content (%) for spray dried dispersions of CFX and TA using three pairs of independent variables: inlet temperature, ethanol ratio and CFX/TA molar ratio.

**Fig. 4:**
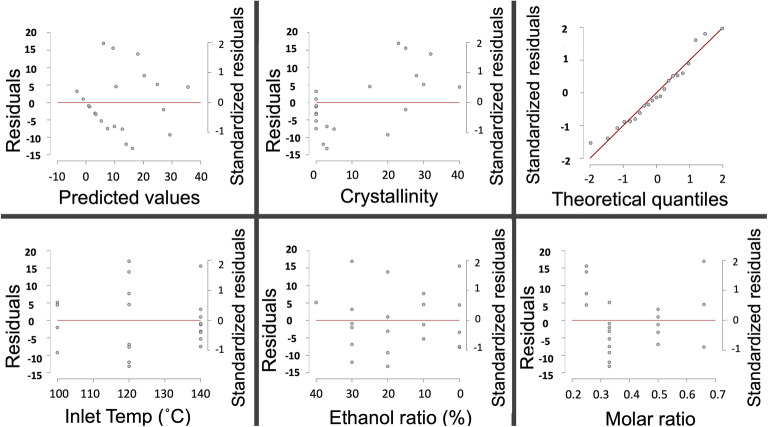
Residual plot analysis of predicted vs residuals, crystallinity vs residuals and theoretical quantiles vs standardized residuals. The bottom three graphs show residuals analysis of spray drying conditions: inlet temperature, ethanol ratio and CFX/TA molar ratio with relation to crystallinity content.

A linear regression analysis was performed with crystallinity content (%) being the dependent variable and the independent variables were: CFX to TA molar ratio, ratio of ethanol to water in solution prior to spray drying and inlet temperature (Fig. 3). Crystallinity content was quantified using the area under the peak for each sample divided by the peaks for 100% crystalline CFX. A total of 22 samples were prepared by spray drying and the data were plotted to find optimum conditions to prepare the dispersions (Fig. 3). The results showed linear correlation between inlet temperature and ratio of ethanol used for spray drying with R^2^ value of 0.95. This indicates that when increasing both of those variables, a linear effect on crystallinity could be observed. Deviation from linearity was observed when the molar ratio was changed (with inlet temperature or ethanol ratio) reflected by R^2^ of around 0.6. To test regression analysis validity to model these data, the predicted values were plotted against residuals which showed random distribution of data points signifying appropriate use of the linear regression (Fig. 4). Likewise, the Q-Q plot indicated good correlation between theoretical quantiles and standardized residuals. Overall, a molar ratio of CFX:TA (1:1) produced co-amorphous dispersions and was selected to form the hybrid microparticles shown in Fig. 1.

**Fig. 5:**
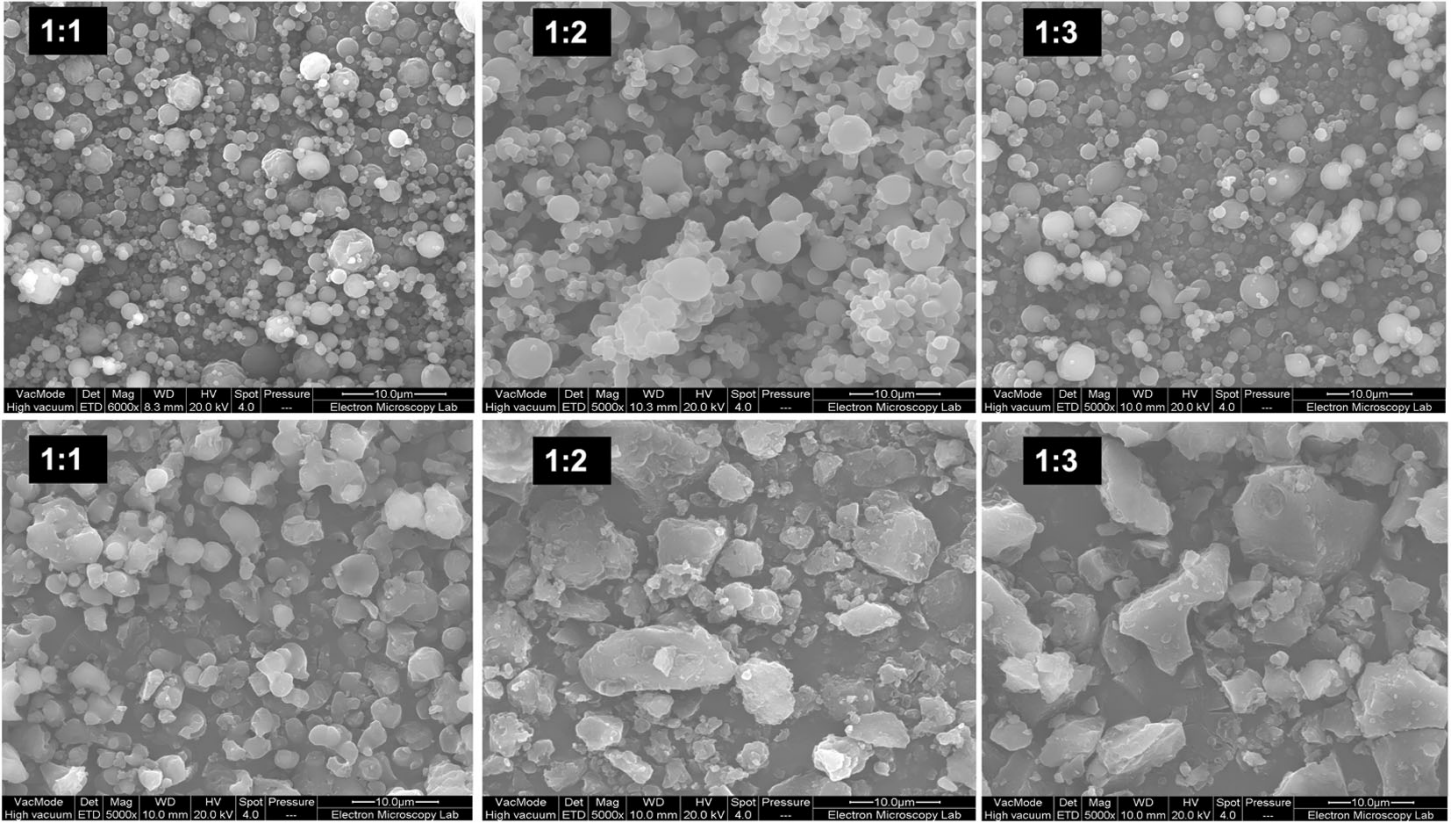
SEM pictures of spray-dried of CFX-TA in different solvent, top samples were prepared in pure water, bottom images refer to dispersions prepared in 10% v/v ethanol in water solution.

As can be seen in (Fig. 5), the particles showed a spherical shape for co-amorphous dispersions of TA to CFX (1:1) with a smooth surface reflecting the amorphous nature of these particles. At CFX: TA 1:2, the shape of the particles remained spherical with some grove like structures started to form at the surface. At CFX: TA 1:3, the particles were also spherical with a uniform distribution of particles size. The impact of solvents composition was more evident where the shape of the particles has changed from spherical to irregular shaped particles with sharp edges especially at higher TA: CFX ratios in line with the XRPD data shown above. Viscosity data (Table I) showed that the viscosity of the TA and CFX solutions varied significantly. Solutions prepared using water showed kinematic viscosity of 0.840–0.872 mm^2^/s while samples prepared using 10% ethanol showed viscosity of 1.096–1.223 mm^2^/s and for solutions prepared using 20% ethanol 1.473–1.582 mm^2^/s. These differences indicate that the viscosity was twice as high compared to the viscosity of water solutions, which may explain the irregular shaped particles formed from ethanolic solutions (Fig. 5). Lower viscosity is expected to favour more spherical structure for the final particles while higher viscosity may result in irregular shaped droplets hence final particles should also be irregular. Higher viscosity could affect the kinetic energy and diffusion of the solvent and therefore the rate of solvent removal is also affected. Although ethanol is more volatile, the higher viscosity combined with lower solubility of CFX led to appearance of crystals as evident in the XRPD data.

**Table I:** Kinematic Viscosity Measurements of Different Ratio with Different Solvents

**CFX:TA**	**Water/ethanol (%)**	**Kinematic viscosity (**mm^2^/s)
1:1	100%	0.84 ± 0.02
1:2	100%	0.86 ± 0.03
1:3	100%	0.87 ± 0.02
1:1	80% /20%	1.52 ± 0.01
1:2	80% /20%	1.53 ± 0.04
1:3	80% /20%	1.47 ± 0.02
1:1	90% /10%	1.19 ± 0.03
1:2	90% /10%	1.22 ± 0.02
2:1	90% /10%	1.09 ± 0.01
1:3	90% /10%	1.11 ± 0.02

### Thermal and Spectroscopic Analysis of CFX/TA Co-Amorphous Dispersions

The main challenge with measuring thermal events was the high melting temperature of CFX which leads to degradation of TA before any melting can be detected. However, thermal analysis showed that the CFX/TA (1:1) dispersions had a glass transition temperature (T_g_) of 153.5°C. This is significantly higher than published data for amorphous CFX (87°C) and tartaric acid (24°C) (22,23). Such high T_g_ compared to pure components suggests that the formed dispersion is co-amorphous salt. This can be demonstrated as deviation from linear correlation based on Gordon Taylor equation.

**Fig. 6:**
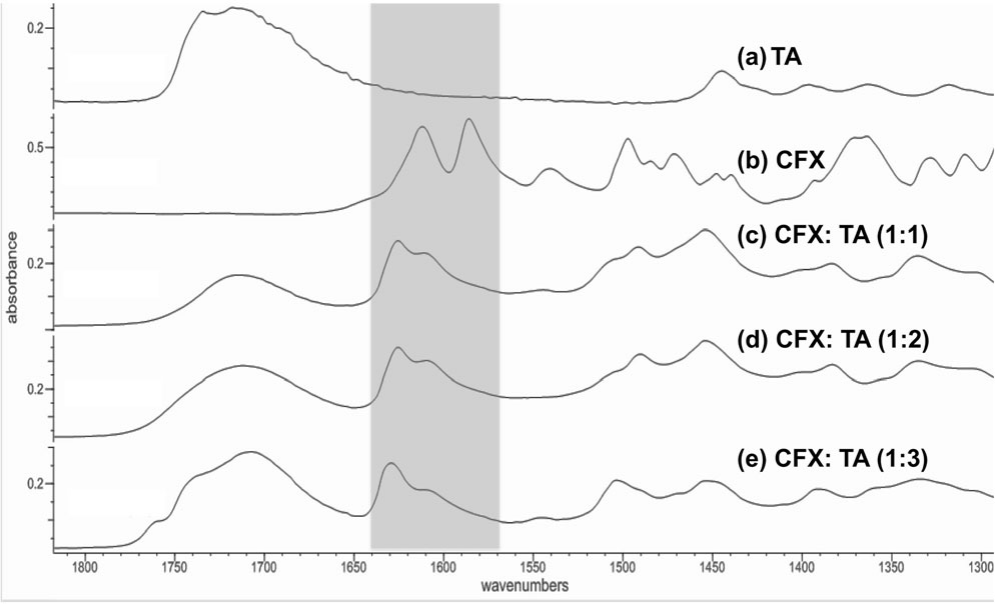
FTIR spectra of CFX, TA and corresponding spray dried dispersions.

In order to understand the nature of intermolecular interactions, FTIR analysis was performed for the spray dried dispersions. As can be seen in Fig. 6, the results showed, in agreement with previous studies, stretching of the carboxyl group in CFX is missing in the region of 1710 cm^−1^ (23,24). It was suggested that a “betainelike structure” exists where electronic effects become more profound leading to the formation of a zwitterion, on the carboxyl (COOH). Slight presence of the zwitterion form could also be seen by the presence of small peaks in the region of 2200–2400 cm^−1^ assigned to piperazine secondary amine. The ionised COO- can still be seen as symmetric and asymmetric vibrations at 1585 and 1375 cm^−1^respectively. The peak at 1611 cm^−1^ corresponds to the C=O stretching in the ketone group. The spectrum for TA showed two peaks at 1717 and 1740 cm^−1^, which correspond to the stretching vibrations of the two carbonyl groups on the carboxylates. Changing the ratio of the TA to CFX has led to the loss of the zwitterion structure as the peak at 1585 cm^−1^ has disappeared indicating formation of free COOH with a potential to form hydrogen bonds. The peak at 1630 cm^−1^ is related to the aromatic carbonyl stretching which has shifted because of the loss of intermolecular hydrogen bonding. The carbonyl group in hydroxylate is more acidic and a stretching could be seen in the region of 1700–1770 cm^−1^ which has also overlapped with the peaks of TA. Altering the ratio of CFX to TA has led to multiple peaks seen within this region indicating increased bonding at the free COOH combined with less tendency for self-association reflected in the notable shift at 1630 cm^−1^. The difference in the pK_a_ values between that of TA and CFX (secondary amine piperazine) is >5 which suggests that the formed structures are co-amorphous tartrate salt. Comparison with CFX hydrochloride salt showed similar peaks with slight variation potentially due to formed hydrogen bonds as well as traces of zwitterionic form especially when ethanol was used.

**Fig. 7:**
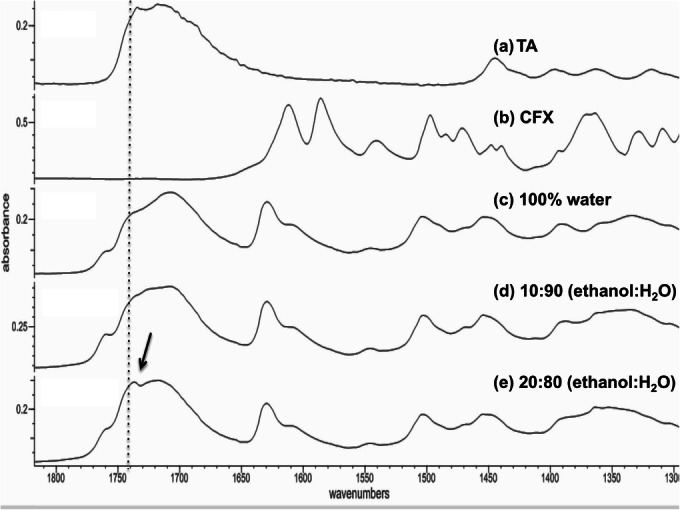
FTIR spectra of CFX, TA and CFX: TA (1:2) prepared using different ethanol/water ratios.

In order to understand whether the presence of ethanol affects the ionisation of CFX and possibly the formation of hydrogen bonds with TA, the spectra for the spray-dried dispersions prepared were collected (Fig. 7). The results showed changes in the peaks positions in the region 1650–1800 cm^−1^ when the solvent was changed from water to a mixture of water and ethanol. Three peaks could be observed at 1715, 1740 and 1760 cm^−1^, which corresponds to stretching vibration of, bound COOH on TA and on CFX. Dispersions prepared from water showed a shift in the peak towards lower frequencies indicating possible intermolecular bonding with less evident shift in the peak position for dispersions prepared using 20% ethanol. Dispersions prepared using similar solvents compositions but using TA: CFX of 1:1 were also analysed. The difference among the prepared samples was less clear which indicates that the impact of solvents was more profound at higher TA: CFX ratio.

### Coating with Silica Coated Silver and NaHCO_3_ and In Vitro Lung Deposition Studies

**Fig. 8:**
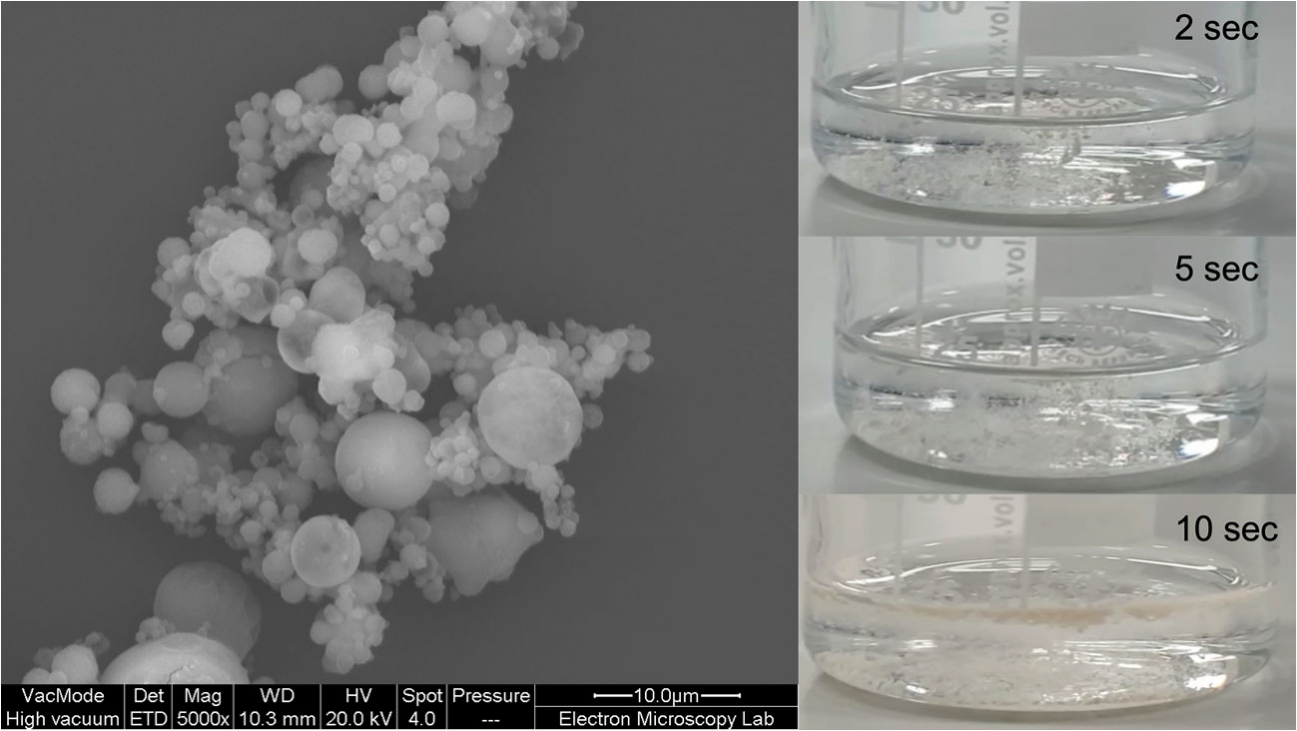
Scanning electron microscopy image of the spray dried hybrid microparticles of CFX/TA with silver coated nanoparticles incorporating NaHCO_3_. The effervescence occurred rapidly within 10 s when suspended in water.

The shell of the hybrid particles comprises and NaHCO_3_ and silver nanoparticles coated with silica to prevent uncontrolled aggregation (Fig. 1). This was achieved following similar method published previously where the silver beads were coated with silica using Stöber method (16). The findings of amorphous form formation were carried forward for the preparation of the hybrid particles containing the silica coated silver core particles. The ratio of CFX:TA (1:1) was used to form the dispersions while the amount of silver was maintained to less than 4–5 ppm in line with our previous findings (16). The rationale for inclusion of sodium bicarbonate was to generate effervescent effect so that to penetrate through the biofilm viscous structure. The particles exhibited similar size and shape as the co-amorphous CFX/TA signifying success of forming the particles (Fig. 8). Strong effervescent effect was initiated in less than 5 s and was completed within 30 s. Generation of CO_2_ adds to the synergy of the particles while attached to the bacterial biofilm allowing the drug to diffuse freely throughout the viscous network (Fig. 8).

Andersen cascade impactor (ACI) simulates the respiratory tract, with the ability to modulate flow rate to simulate different inspiratory flow rates. The induction port represents the throat with a 90° angle. The ACI body includes 8 stages of progressively smaller nozzle diameters, and therefore, progressively smaller aerodynamic particle cut-off sizes. This design mimics the upper and lower respiratory tracts and pulmonary deposition of inhaled formulations, with a micro-orifice collector (MOC) acting as a final impaction stage for extremely small particles. As can be seen in Table II, The spherical hybrid co-amorphous spray dried particles (that contained silver and sodium bicarbonate) showed a mean aerodynamic diameter (MMAD) of 3.33 μm and GSD of 2.98 μm (Table II). Fine-particle dose (FPD) is defined as the dose of the inhaled drug particles with an aerodynamic diameter < 5 μm while fine particle fraction (FPF) is the ratio of FPD to the total emitted dose (ED). As can be seen, emitted dose was 23.46 mg (78%) while FPD was 19.83 mg (85%). Particles prepared with silica nanoparticles (without silver or sodium bicarbonate) showed MMAD of approximately 3.8 μm and GSD 2.6 μm. The emitted dose was 24.14 mg (79%) and FPD of 20 mg (82%). Particles prepared with silica coated silver showed slightly higher MMAD of or 4.8 μm with GSD of 2.2 μm while the ED and FPD were 18.2 mg (61%) and 14.8 mg (81%), respectively. When compared with crystalline CFX, ED was significantly lower of around 4.5 mg (15%) reflecting significant loss and poor deposition. There was less variation in the coarse particle fraction (CFP) among the samples with a median value of around 19%. The extra fine particle fraction (EFPF) showed higher variation with the CFX and CFX/TA physical mixture showing the lowest values of approximately 2.5–6.6% compared with the hybrid particles showing EFPF of 18%. The fine particle fraction (FPF) was less discriminative among the samples with highest values were found for the hybrid particles of 84.5%. When comparing the MMAD, it becomes clear that the hybrid particles exhibited optimum value of 3.33 μm when compared with CFX (8.05 μm) and CFX/TA physical mixture (5.3 μm). Particles in the aerodynamic size range from 1.5–5 μm were shown to be optimal, as particles <1.0 μm are likely to be exhaled again while those >5 μm may impact on the oropharynx (25).

**Table II:** Deposition Parameters of CFX Dispersions Assessed Using Andersen Cascade Impactor

**Formulation (All contain CFX/TA)**	**MMAD (**μm**)**	**GSD (μm)**	**ED (mg)**	**FPD (mg; <5 μm)**	**FPF (%; <5 μm)**	**CPF (%; >5 μm)**	**EFPF (%; <1 μm)**
* Silica coated Ag NPs + NaHCO_**3**_	3.33 ± 0.12	2.98 ± 0.47	23.46 ± 0.56	19.83 ± 0.89	84.5 ± 1.78	15.5 ± 1.78	18.04 ± 0.03
* Silica coated Ag NPs	4.82 ± 0.48	2.24 ± 0.05	18.2 ± 1.54	14.8 ± 1.7	80.1 ± 2.47	19.03 ± 2.47	9.6 ± 1.68
* Silica (without Ag)	3.82 ± 0.35	2.59 ± 0.25	24.14 ± 0.09	20.01 ± 0.97	82.9 ± 3.7	17.09 ± 3.7	16.84 ± 0.96
**CFX/TA 1:1 physical mixture**	5.3 ± 0.09	4.52 ± 0.93	4.61 ± 0.14	3.65 ± 0.05	79.2 ± 1.3	20.8 ± 1.3	2.5 ± 0.72
**CFX (crystalline)**	8.05 ± 0.13	2.25 ± 0.42	4.35 ± 0.11	3.24 ± 0.23	74.37 ± 0.35	25.62 ± 0.35	6.62 ± 0.3

Abbreviations: MMAD (mass median aerodynamic diameter), GSD (geometric standard deviation), ED (Emitted Dose), FPD (Fine particle dose), FPF (Fine particle fraction), CPF (Coarse particle fraction) and EFPF (Extra-fine particle fraction)

*Indicates significant difference in ED and FPD, when compared to CFX (*p* < 0.05, unpaired Student’s t test)

### Assessment of Antimicrobial Activity against *P. aeruginosa*

**Fig. 9:**
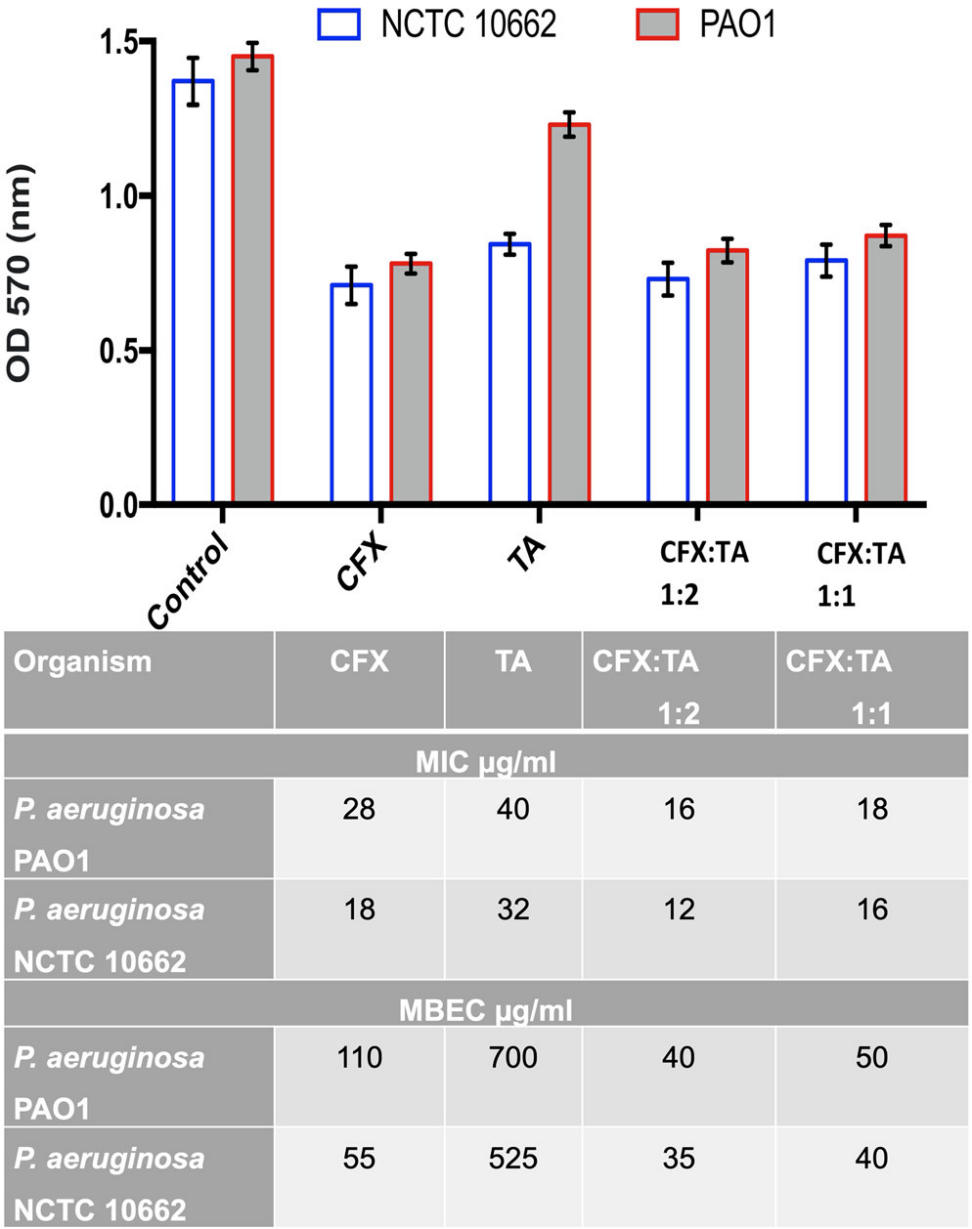
Quantification of biofilm formation by *P. aeruginosa* PAO1 and NCTC 10662 along with MIC and MBEC.

Assessment of antimicrobial activity against *P. aeruginosa* was performed for all formulations containing CFX and TA (1:1 and 1:2). The main characteristic difference between the two strains of *P. aeruginosa* used in this study was the level of mucoidy of the biofilms formed. The mucoidy is dictated by alginate composition of the biofilm. *P. aeruginosa* PAO1 in this respect showed a higher level of mucoidy compared to *P. aeruginosa* NCTC 10662 as NCTC 10662 is considered a non-mucoid strain. The MIC and MBEC of CFX:TA 1:1, 1:2 and pure components are shown in Fig. 9. Higher levels of CFX were required to inhibit *P. aeruginosa* biofilms as opposed to planktonic cells. CFX MIC for *P. aeruginosa* PAO1 was 28 μg/mL, whilst the MBEC was 110 μg/mL. This represents over a fivefold increase in CFX concentration required for treatment of biofilms as opposed to planktonic cells. Notably, MIC for *P. aeruginosa* for CFX:TA (ratios 1:1 and 1:2) the MIC was found to be 18 μg/mL and 16 μg/mL, respectively. Biofilm formation of both *P. aeruginosa* strains was significantly inhibited by CFX:TA formulations in comparison to the control (Fig. 9). CFX:TA formulations were also more effective compared to CFX at biofilm formation inhibition (Fig. 9). The stated molar ratios of 1:1 contains approximately 68% *w*/w of CFX and 32% w/w TA. Hence, this suggests that the 1:1 mixtures had MBEC of around 3.3 times than CFX alone. On the other hand, CFX:TA molar ratios of 1:2 contains approximately 52.5% w/w of CFX and 47.5% w/w TA. The latter imply superior MBEC of around 5.5 times higher than CFX alone.

**Fig. 10:**
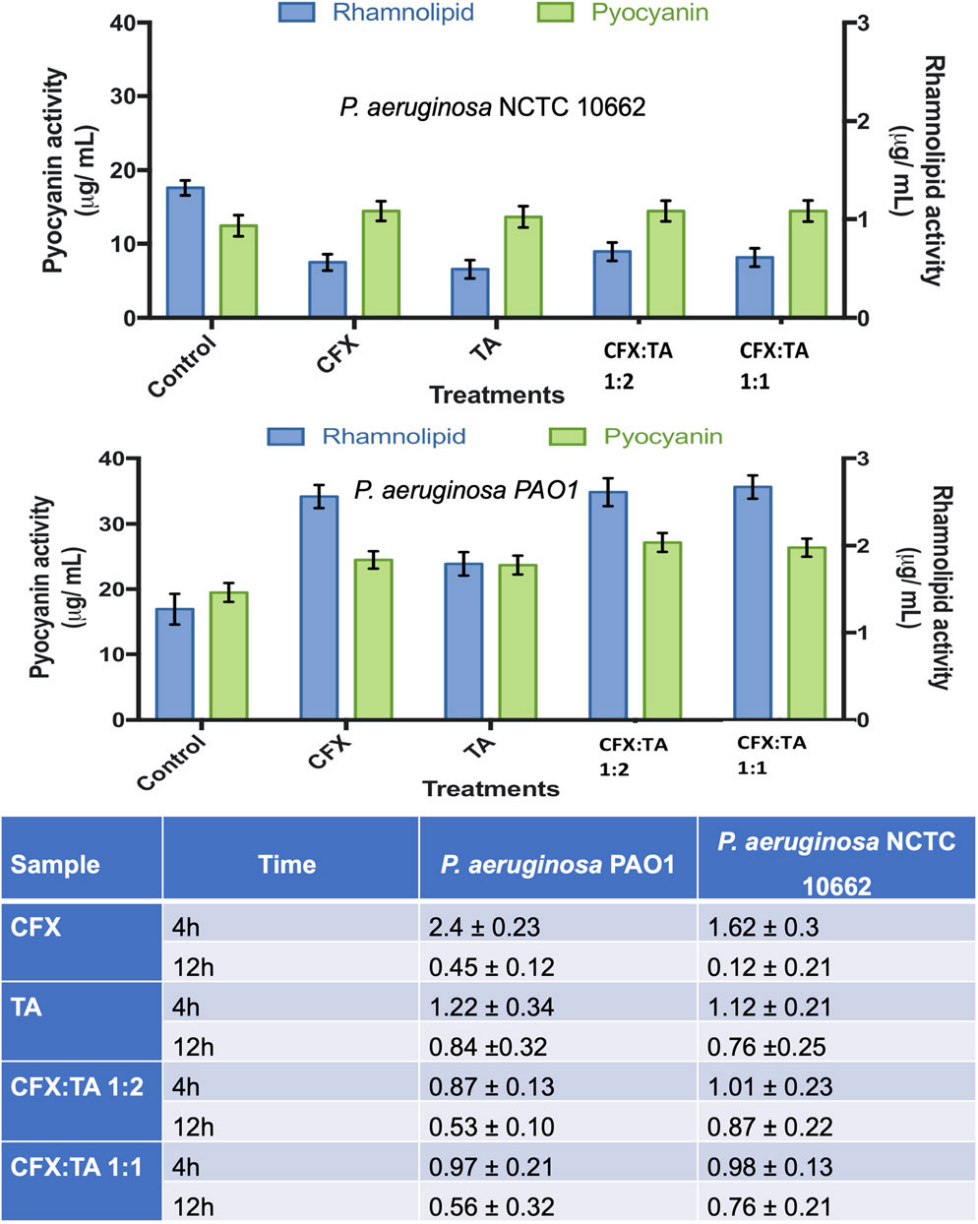
Quantification of pyocyanin and rhamnolipid activity by *P. aeruginosa* NCTC 10662 and *P. aeruginosa* PAO1 with and without treatment. Cell viability of sessile cells present in the biofilm by plating expressed as Log 10 cfu/ ml.

These results were further supported by the microbial cell count. It is clearly evident from Fig. 10, that the formulations used were more effective in reduction of microbial cell count of *P. aeruginosa*, especially in the case of *P. aeruginosa* PAO1, however the presence of a thicker biofilm also indicates the retention of pyocyanin and rhamnolipid (Fig. 10). Pyocyanin and rhamnolipid are two of the virulence factors of *P. aeruginosa* that aid in its pathogenicity. However, the formulations used are clearly effective in significantly reducing the production of rhamnolipid as seen in the case of *P. aeruginosa* NCTC 10662 (Fig. 10). Pyocyanin, a phenazine siderophore is a secondary metabolite produced by *P. aeruginosa* which possesses antibacterial activity. The antibacterial activity of pyocyanin is vital and prevalent in *P. aeruginosa* in the presence of antagonists as it aids in the survival of the colony of bacterial cells. The activity of pyocyanin is highlighted in Fig. 10, which shows that presence of CFX and TA independently or in formulations did not reduce pyocyanin production due to said “survival” mechanism of *P. aeruginosa.*

## Discussion

Co-amorphous dispersions are homogenous mixtures that eliminate the use of carrier particles for pulmonary drug delivery. Encapsulation of the co-amorphous salt inside a layer of silver coated nanobeads and NaHCO_3_ is a novel approach for enhancing stability and augment antibacterial properties due to silver and effervescent effect. Our results showed there was no signs of premature effervescence signifies that the TA component did not react with NaHCO_3_. In order to design this system, linear regression analysis was undertaken to optimise spray drying conditions.

Factors that predispose crystallisation depend on molecular interactions, which are typically stronger than the solvation forces that exist between water and solutes (26,27). In the CFX and TA system the most likely intermolecular interactions are ionic and hydrogen bonds (28). When CFX was mixed with TA solution, the solubility was enhanced as a result of protonation of CFX and associated deprotonation of TA. The net outcome would be equilibrium between the two species where CFX becomes more soluble as long as the protonation-deprotonation balance is maintained (29,30). During spray drying, the process involves rapid removal of water which is associated with crust formation (solid shell) with water in the core of the droplet (31). This process is critical for forming co-amorphous CFX/ TA, as at this stage CFX can precipitate/crystallise out as a separate phase. It was shown before that the evaporation rate of the solvents can significantly affect the extent of intermolecular interactions during spray drying (32). Using ethanol reduces ionic strength and hence less likely to form the tartrate salt. More importantly, ethanol serves as an anti-solvent as CFX is practically insoluble in ethanol and therefore precipitation may occur. The impact of the solvent and the used inlet temperature was correlated and a balance between both was essential to ensure co-amorphous salt was formed. These two factors were significantly affected by alteration of the molar ratio as shown in regression and residuals analysis. The impact of spray drying conditions was reflected in viscosity data where ethanol solutions were more viscous which likely to promote crystallisation. Incorporation of the silver coated silica with the NaHCO_3_ had minimum impact on the morphology as was seen by the SEM images. The formed effervescent effect was very rapid indicating successful design of the particles. The formed particles exhibited optimum MMAD and GSD values demonstrating the potential to target bacterially inhabited regions within the lungs. There have been limited studies exploring toxicity of inhaled silver nanoparticles, however with the extremely low amounts of silver used in our study, the amount delivered is unlikely to cause harm. For example, homeopathic silver preparations can contain 10–15 μg of silver which is within the range used in this study (16).

The biofilm inhibition and virulence factors assays were performed using the concentrations obtained from MIC and MBEC data. These implied that the independent data points (MIC & MBEC) obtained for CFX and TA are used as positive controls and the effect of the samples will be compared against them. For example, the data showed that MBEC of CFX:TA (40 μg/mL) against *P. aeruginosa* PAO1 which contains ~52.5% CFX (~21 μg/mL) + 47.5% TA (19 μg/mL) is more potent compared to independent values for CFX (MBEC of 110 μg/mL) and TA (MBEC of 700 μg/mL). So approximately 1/5th of the total CFX concentration is more potent when used in combination with TA compared to CFX and TA alone. The zwitterion structures of CFX were altered in the CFX:TA,as could be seen in FTIR, and could possibly enhance the activity of CFX. The transitional appearance of the cation betaine like structures (upon dissociation of the salt) and release of CO_2_ gas (upon reaction with NaHCO_3_) are likely to play a pivotal role in enhanced synergistic effect of these co-amorphous salts.

**Fig. 11:**
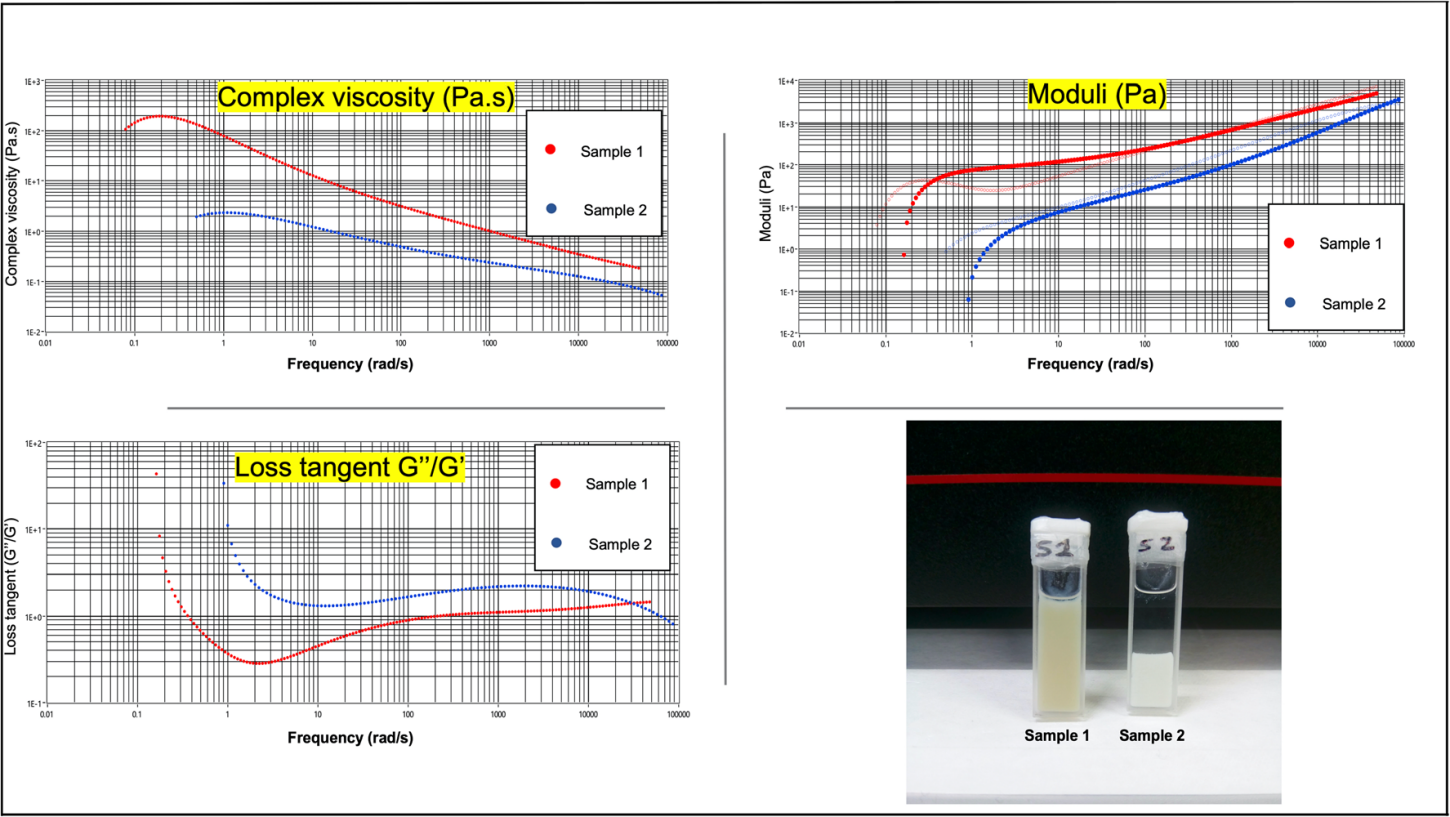
Complex viscosity (Pa.s), moduli (Pa) and loss tangent (G”/G’) of sample 1: spray dried hybrid microparticles with silver nanobeads shell and sample 2: spray dried microparticles without the silver.

Microrheology can be used to understand the impact of the particles on the integrity of the biofilms. Microrheology studies were carried out using glycerol at the suspending medium. Glycerol to simulate bacterial biofilms was used in previous studies to simulate viscous contribution of the media (33,34). This is to help understand the impact of particle engineering and inclusion of the dense silver particles. The prepared particles were suspended in same volume of glycerol and changes were monitored for up to 30 min by which different rheological parameters were recorded and analysed. As can be seen in Fig. 11, the sample which contained the hybrid microparticles remained suspended. On the contrary, the sample which contained no silver has settled rapidly which indicates different viscoelastic properties. Examination of rheological properties reveals higher complex viscosity of sample 1 (with silver) than sample 2 (no silver). The loss moduli were also different with higher loss modulus for sample 1. This thixotropic effect is likely to cause additional synergistic effect in disrupting the highly viscous biofilm structures. These results suggest novel role for the prepared particles via disruption of the viscoelastic properties in addition to the effervescence generated due to interactions between NaHCO_3_ and TA.

## Conclusion

In summary, this study represents novel approach in carrier-free, particle engineering of DPI formulations. The preparation of CFX:TA formulations by spray drying is a straightforward process, offering a novel co-amorphous salt without the use of carriers. This formulation achieved excellent in vitro deposition performance to target the conducting zone where *P. aeruginosa* biofilms are most problematic to eradicate. Most importantly, the use of TA as a co-former enabled this novel formulation to have a potent, synergistic effect against *P. aeruginosa* biofilms. These CFX:TA formulations could provide the first inhaled CFX formulation, and offer an additional treatment option for clinicians faced with treating resistant *P. aeruginosa* infection.

## Abbreviations

CFX: ciprofloxacin
CPF: (Coarse particle fraction)
DOE: design of experiments
ED: (Emitted Dose)
EFPF: (Extra-fine particle fraction)
FPD: (Fine particle dose)
GSD: (geometric standard deviation)
MMAD: (mass median aerodynamic diameter)
PFF: (Fine particle fraction)
TA: tartaric acid
